# Ultrasound-Targeted Microbubble Destruction (UTMD) Assisted Delivery of shRNA against PHD2 into H9C2 Cells

**DOI:** 10.1371/journal.pone.0134629

**Published:** 2015-08-12

**Authors:** Li Zhang, Zhenxing Sun, Pingping Ren, Robert J. Lee, Guangya Xiang, Qing Lv, Wei Han, Jing Wang, Shuping Ge, Mingxing Xie

**Affiliations:** 1 Department of Ultrasound, Union Hospital, Tongji Medical College, Huazhong University of Science and Technology, Hubei Province Key Laboratory of Molecular Imaging, Wuhan, 430022, PR China; 2 Division of Pharmaceutics, College of Pharmacy, The Ohio State University, Columbus, Ohio, 43210, United States of America; 3 School of Pharmacy, Tongji Medical College, Huazhong University of Science and Technology, Wuhan, 430022, PR China; 4 The Heart Center, St. Christopher's Hospital for Children/Drexel University College of Medicine, Philadelphia, Pennsylvania, United States of America; University of Cincinnati, College of Medicine, UNITED STATES

## Abstract

Gene therapy has great potential for human diseases. Development of efficient delivery systems is critical to its clinical translation. Recent studies have shown that microbubbles in combination with ultrasound (US) can be used to facilitate gene delivery. An aim of this study is to investigate whether the combination of US-targeted microbubble destruction (UTMD) and polyethylenimine (PEI) (UTMD/PEI) can mediate even greater gene transfection efficiency than UTMD alone and to optimize ultrasonic irradiation parameters. Another aim of this study is to investigate the biological effects of PHD2-shRNA after its transfection into H9C2 cells. pEGFP-N1 or eukaryotic shPHD2-EGFP plasmid was mixed with albumin-coated microbubbles and PEI to form complexes for transfection. After these were added into H9C2 cells, the cells were exposed to US with various sets of parameters. The cells were then harvested and analyzed for gene expression. UTMD/PEI was shown to be highly efficient in gene transfection. An US intensity of 1.5 W/cm^2^, a microbubble concentration of 300μl/ml, an exposure time of 45s, and a plasmid concentration of 15μg/ml were found to be optimal for transfection. UTMD/PEI-mediated PHD2-shRNA transfection in H9C2 cells significantly down regulated the expression of PHD2 and increased expression of HIF-1α and downstream angiogenesis factors VEGF, TGF-β and bFGF. UTMD/PEI, combined with albumin-coated microbubbles, warrants further investigation for therapeutic gene delivery.

## Introduction

Despite its great potential, the lack of safe, efficient and specific delivery systems remains the biggest roadblock to the clinical applications of gene therapy. Viruses can be efficient as delivery vehicles. However, they are often associated with serious problems such as immunogenicity and cytotoxicity [1–2]. The efficiency of nonviral vectors is usually poor [3–4]. Therefore, more efforts are needed to make improvements on nonviral vectors. Among novel strategies under investigation, ultrasound-targeted microbubble destruction (UTMD) has shown to be particularly promising for enhancing gene/drug delivery [5–7].

The biological effects of UTMD can be affected by the compositions of the microbubbles and the plasmid complexes, as well as parameters of US irradiation. Recently, UTMD combined with polyethylenimine (UTMD/PEI) was shown to have excellent transfection efficiency [8–9].

RNA interference (RNAi) is a process through which double-stranded RNAs (dsRNA) induce efficient sequence-specific post-transcriptional silencing of genes. RNAi has been a useful tool of gene function analysis [10]. It can be introduced in the form of a plasmid that codes for a short hairpin RNA (shRNA). Prolyl hydroxylase domain-containing protein 2 (PHD2) is encoded by the EGLN1 gene. PHD2 is the primary regulator of hypoxia-induced factor-1α (HIF-1α) and plays important roles in human diseases. Research suggested that silencing PHD2 gene can prevent HIF-1α protein catabolism and HIF-1α protein increase can in turn induce expression of angiogenesis factor, such as VEGF, FGF-2 and TGF-β [11–12]. In addition, increase in expression of HIF-1α gene has been shown to prompt compensatory adaption of ischemic myocardium [10].

In this article, UTMD/PEI mediated gene transfection was investigated and the US parameters were optimized. Furthermore, the biological effects of PHD2 shRNA were investigated in H9C2 cells.

## Materials and Methods

### Cell cultures

Rat cardiac H9C2 cells purchased from Boster Biological Engineering Co.,Ltd. China, were grown in Dulbecco’s modified Eagle’s medium (DMEM, Hyclone, Logan, UT, USA) with 10% fetal bovine serum, penicillin (100 U/ml)and streptomycin (100 U /ml) (Life Technologies, Carlsbad, CA, USA). The cells were cultured at 37°C in a humidified incubator and an atmosphere of 5% (v/v) CO_2_.

### Preparation of plasmid DNA, microbubbles and transfection complexes

pEGFP-N1 and shPHD2-EGFP (target sequence: TCGTGCCGTGCATGAA CAA) plasmids were purchased from Shanghai Genechem. Co., Ltd. China. The plasmids were amplified in *E*.*coli* and purified using a plasmid DNA purification kit (Qiagen GmbH, Hilden, Germany). DNA concentration, measured by absorption at 260nm, was 1 mg/ml.

The Perfluoropropane-Albumin Microsphere (PFPAM) (Hunan Zhongnan Kelun Pharmaceutical Co., Ltd.) used in our experiment was an albumin-shelled US contrast agent composed of microbubbles filled with perfluoropropane gas. The microbubbles were 3.04 to 3.56 μm in mean diameter. The PFPAM contains 5.18–6.08 × 10^8^ microbubbles per ml.

JetPEI (Polyplus, Paris, France) and pEGFP-N1 or PHD2-shRNA were mixed in 150 mM NaCl at a volume ratio of 2:1 (N/P = 5) according to the manufacturer's directions and incubated for 20 min at room temperature. Then, plasmid DNA or PEI/DNA complexes and PFPAM were gently mixed in Opti-MEM (Life Technologies, USA) at a final volume of 500 μl to form the transfection complexes. The transfection complexes were incubated for 15 min before being used in transfection.

### Cellular transfection with PEI/DNA complexes with ultrasonic exposure

Before experiment, H9C2 cells were cultured in 24-well plates (Becton Dickinson, USA) at 1.0×10^5^ cells/per well overnight. For US exposure studies, the parameters examined were acoustic intensity (AI), microbubble concentration (MC), exposure time (ET), and plasmid concentration (PC).The cells were divided into the following five groups (n = 6): A: untreated, B: treated with 5 μg pEGFP-N plasmid DNA, C: treated with PEI/DNA complexes (including 5 μg pEGFP-N and 10 μl PEI), D: treated with PEI/DNA complexes with ultrasonic exposure (AI 1.5 W/cm^2^, MC 300 μg/ml, PC 5 μg/ml and ET30 s), E: treated with PEI/DNA complexes plus PFPMS with ultrasonic exposure (AI 1.5 W/cm^2^, MC 300 μg/ml, PC 5μg/ml and ET30 s). For US irradiation, after the addition of plasmid or transfection complexes, a sterile US probe (Sonitron 2000V, Japan) was inserted directly into the bottom of the 24-well plate.

### Optimization of ultrasonic irradiation parameters

A series of US parameters AI, MC, ET and PC were evaluated in the optimal transfection group (one group of above five groups): US frequency, 1 MHz; duty cycle, 20%; AIs: 0.5, 1.0, 1.5, and 2.0 W/cm^2^; MCs, 100 μl/ml, 200 μl/ml, 300 μl/ml and 400 μl/ml; ET, 15, 30, 45 and 60 s; and PCs, 5, 10, 15, and 20 μg/ml. After the addition of PEI/DNA transfection complexes plus PFPMS in 500 μl, a sterile US probe (Sonitron 2000V, Japan) was inserted directly into the bottom of the 24-well plate and the cells were exposed with US.

### Transfection of EGFP or shPHD2-EGFP plasmid into normoxic and hypoxic H9C2 cells by UTMD/PEI

Normoxic H9C2 cells were cultured under normal condition (5% CO2, 21% O2, and 74% N2) in a humidified incubator at 37°C. Meanwhile, hypoxic H9C2 cells were cultured in a triple gas incubator with hypoxic settings (5% CO2, 1% O2, and 94% N2). In this part of the study, cells were divided into the following four groups (n = 6): Normoxic & EGFP: normoxic H9C2 cells transfected with EGFP plasmid; Normoxic & shPHD2-EGFP: normoxic H9C2 cells transfected with shPHD2-EGFP plasmid; Hypoxic & EGFP: hypoxic H9C2 cells transfected with EGFP plasmid; Hypoxic & shPHD2-EGFP: hypoxic H9C2 cells transfected with shPHD2-EGFP plasmid.

### Assessment of H9C2 cell viability

Cell viability was measured at 48 h after the transfection, using Cell Counting Kit-8 (CCK-8) according to the manufacture’s protocol (Dojindo, Japan). The cells were suspended and transferred into 96-well plates (100 μl, 1×10^4^ per well) (Falcon, USA). Then, 10 μl CCK8 was added to each well. After 2 h incubation, the absorbances of the plates were measured at 450 nm using a microplate reader (BioRad, USA). Cell viability (%) was calculated according to the following equation: Cell viability(%) = (A_sample_ / A_control_) 100%. Each reading was performed 3 times.

### Assessment of EGFP Expression

At 12 h, 24 h, 48 h and 72 h after transfection, H9C2 cells in 24-well plates were evaluated for EGFP expression using a fluorescence inverted microscope. EGFP transfection efficiency was measured also by flow cytometry. First, the transfected H9C2 cells were harvested using 0.25% Trypsin-EDTA and washed twice using PBS. Secondly, the concentration of the resuspended H9C2 cells was adjusted to approximately 1.0×10^6^ cells per ml. Finally, DNA transfection efficiency (the number of cells expressing EGFP per number of cells in total) was determined by assessing the EGFP expression using flow cytometer at an excitation wavelength of 488 nm.

### Gene quantification and Western-blot

To confirm the shPHD2 knocking down efficiency, PHD2, HIF-1α, VEGF, bFGF and TGF-β1mRNA and protein levels were detected by qRT-PCR and Western-blot respectively.

Total RNA was isolated from H9C2 cells (Normoxic & EGFP, Normoxic & shPHD2-EGFP, Hypoxic & EGFP and Hypoxic & shPHD2-EGFP group) using TRIZOL Reagent (Invitrogen, USA). Real-time RT-PCR analysis of PHD2, HIF-1α, VEGF, bFGF and TGF-β1 mRNA levels was performed using One Step SYBR Prime Script RT-PCR kit (TaKaRa, China) according to the manufacturer’s instructions. The flowing primers were designed for real-time RT-PCR: β-actin, forward 5’-TGACGTGGACATCCGCAAAG-3’, reverse: 5’-CTGGAAGGTGGAC AGCGAGG-3’; PHD2, forward: 5'-TACAGGATAAACGGCCGAAC-3', reverse: 5'-TTGGGTTCAATGTCAGCAAA-3'; HIF-1α, forward: 5'-CGCAGTGTGGCTAC AAGAAA-3', reverse: 5'-TAAAT TGAACGGCCCAAAAG-3'; TGF-β1, forward: 5‘-G TCAACTGTGGAG CAACACG-3’, reverse: 5‘-ACTGAAGCGAAAGCCCTGTA-3’; VEGF, forward: 5’-GCCCATGAAGTGGTGAAGTT-3’, reverse: 5’-CTATGTGCTG GCTTTGGTGA-3’; bFGF, forward: 5‘-GAACCGGTACCTGGCTATGA-3’, reverse: 5’-ACTGCCCAGTTCGTTTCAGT-3’. All the primers were synthesized by Shanghai Genechem Co., Ltd (Shanghai, China). Amplification conditions were as follows: 42°C for 5 min, 95°C for 10 s, followed by 40 cycles at 95°C for 5 s, and 60°C for 30 s. The relative quantities of PHD2, HIF-1α, VEGF, bFGF and TGF-β1 mRNA were determined by calculating the values of 2^-ΔΔCT^, with each sample being normalized to the expression level of β-actin. The experiment was performed in triplicate.

Total protein was extracted from H9C2 cells using a RIPA buffer with protease and phosphatase inhibitors and used for Western-blot. The following primary antibodies for targeted molecules were diluted at 1:100–1:1000 and were purchased from Santa Cruz (VEGF, catalog # sc-13083 and TGF-β1, catalog # sc-146), Abcam (bFGF, catalog # ab8880) and Bioworld (PHD2, catalog # BS6184 and HIF-1α, catalog # BS3514). An HRP Linked Rabbit IgG (Santa Cruz, catalog # sc2793) was used as a secondary antibody. GAPDH (glyceraldehyde-3- phosphate dehydrogenase) antibody (Santa Cruz, catalog # sc25778) was used as a loading control. The density of the respective bands was quantitated using a densitometer with Alpha View Software for Fluor Chem Systems (ProteinSimple).

### Statistical analysis

All experiments were performed at least three times and representative results are shown. All results are summarized as the mean ± standard deviation (SD). ANOVA and independent two sample t-test were used to evaluate statistical significance of differences between different treatment groups. The data were analyzed using SPSS 19.0 software. *P*<0.05 was considered statistically significant.

## Results

### EGFP Expression in H9C2 Cells

**Fig 1:**
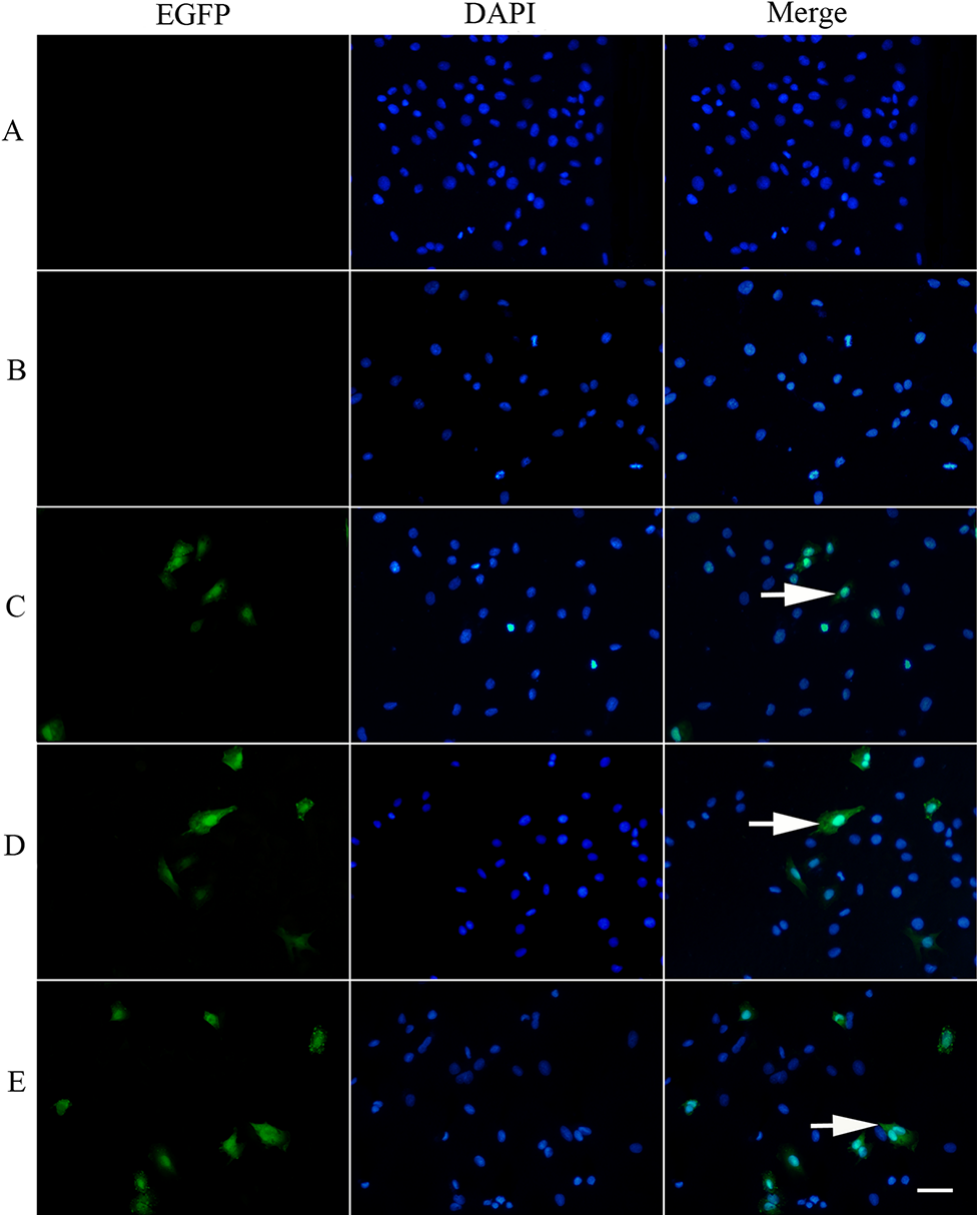
EGFP expression in H9C2 Cells of Group A-E at 48 h post-transfection (200×). (A): untreated group; (B): treated with pEGFP-N plasmid DNA; (C): treated with PEI/DNA complexes; (D): treated with PEI/DNA complexes with ultrasonic exposure; (E): treated with PEI/DNA complexes plus PFPMS with ultrasonic exposure.

After initial transfection, the EGFP protein expression in the H9C2 cells was detected by a fluorescence microscope. At 12 h after transfection, green fluorescence was seen in a few H9C2 cells in group C—E but not in group A—B. The green cell count increased at 24 h and attained the maximum at 48 h. The green fluorescent cells were the most numerous and bright in the group E (Fig 1).

### The Effect of US Parameters on Transfection Efficiency and H9C2 cell Viability

**Fig 2:**
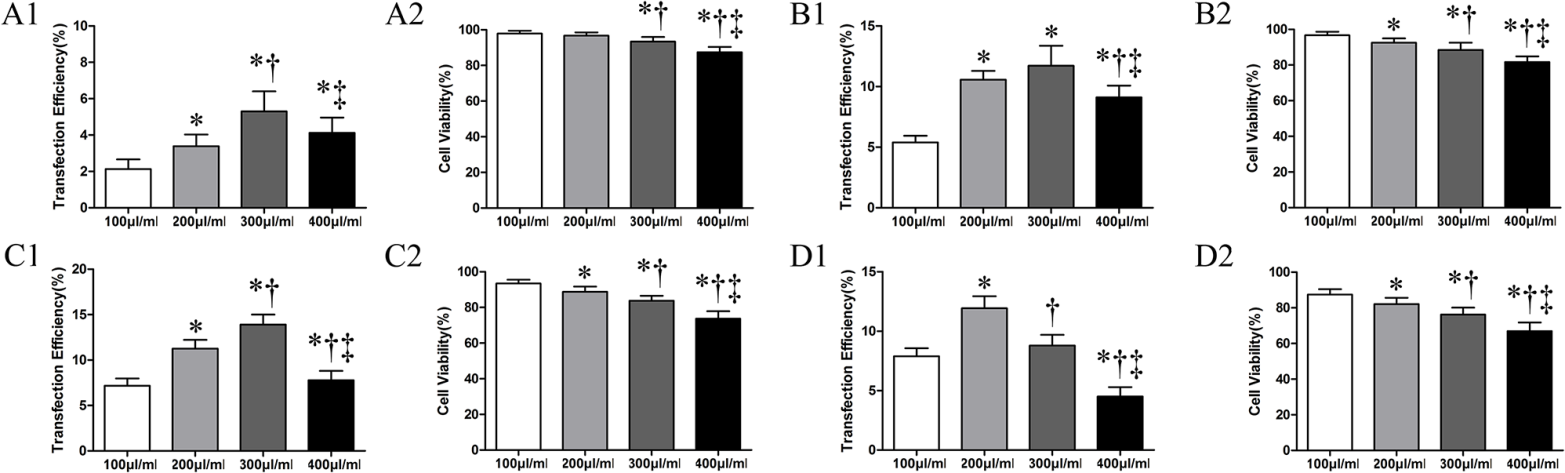
Effects of AI and MC on cell transfection efficiency and cell viability. (A1-A2): transfection efficiency and cell viability at different MC (100 μl/ml, 200 μl/ml, 300 μl/ml and 400 μl/ml) when AI was 0.5W/cm^2^. (B1-B2): transfection efficiency and cell viability at different MC (100 μl/ml, 200 μl/ml, 300 μl/ml and 400 μl/ml) when AI was 1.0 W/cm^2^. (C1-C2): transfection efficiency and cell viability at different MC(100 μl/ml, 200μl/ml, 300μl/ml and 400μl/ml) when AI was 1.5W/cm^2^. (D1-D2) transfection efficiency and cell viability at different MC (100 μl/ml, 200 μl/ml, 300 μl/ml and 400 μl/ml) when AI was 2.0 W/cm^2^. Indication: Symbols indicate significant differences (*P* < 0.05) from the 100μg/ml. (*), from 200μg/ml (†) and from 300μg/ml (‡)respectively.

During transfection, the US probe was set different parameters with the frequency fixed at 1 MHz, EGFP transfection efficiency initially increased with increases of AI and MC. However, when AI was over 1.5 W/cm^2^ and MC over 300 μl/ml, the expression of EGFP did not increase significantly or actually reduced. In addition, cells viability decreased (*P*<0.05) (Fig 2).

**Fig 3:**
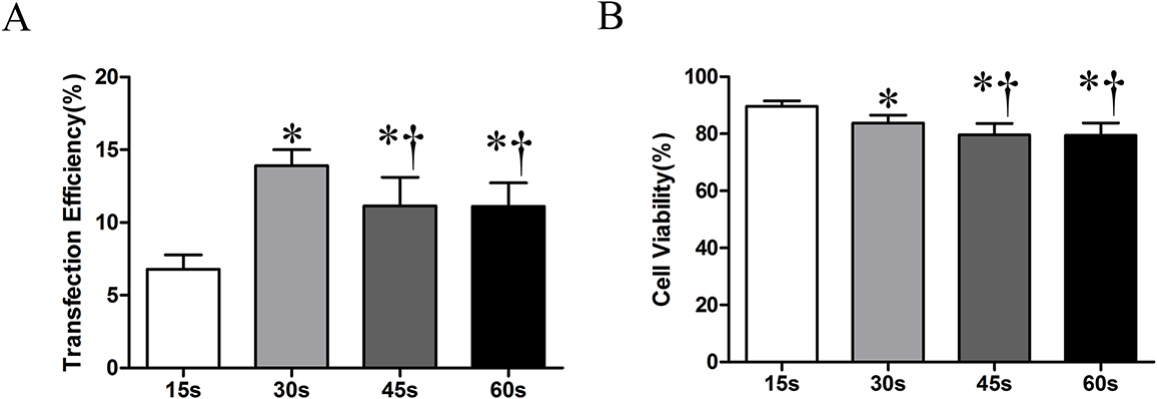
The effect of ET on cell transfection efficiency and cell viability. (A): transfection efficiency at different exposure time; (B): cell viability at different exposure time. Indication: Symbols indicate significant differences (*P*< 0.05) from ET of 15 s (*) or 30 s (†).

Next, AI(1.5 W/cm^2^), MB(300 μl/ml)、PC (5 μg/ml) were fixed. ET was varied (15 s、30 s、45 s、60 s). Maximum transfection efficiency was obtained when the ET was 30 s, as shown in Fig 3. Cell viability was slightly reduced with increase in ET (Fig 3).

**Fig 4:**
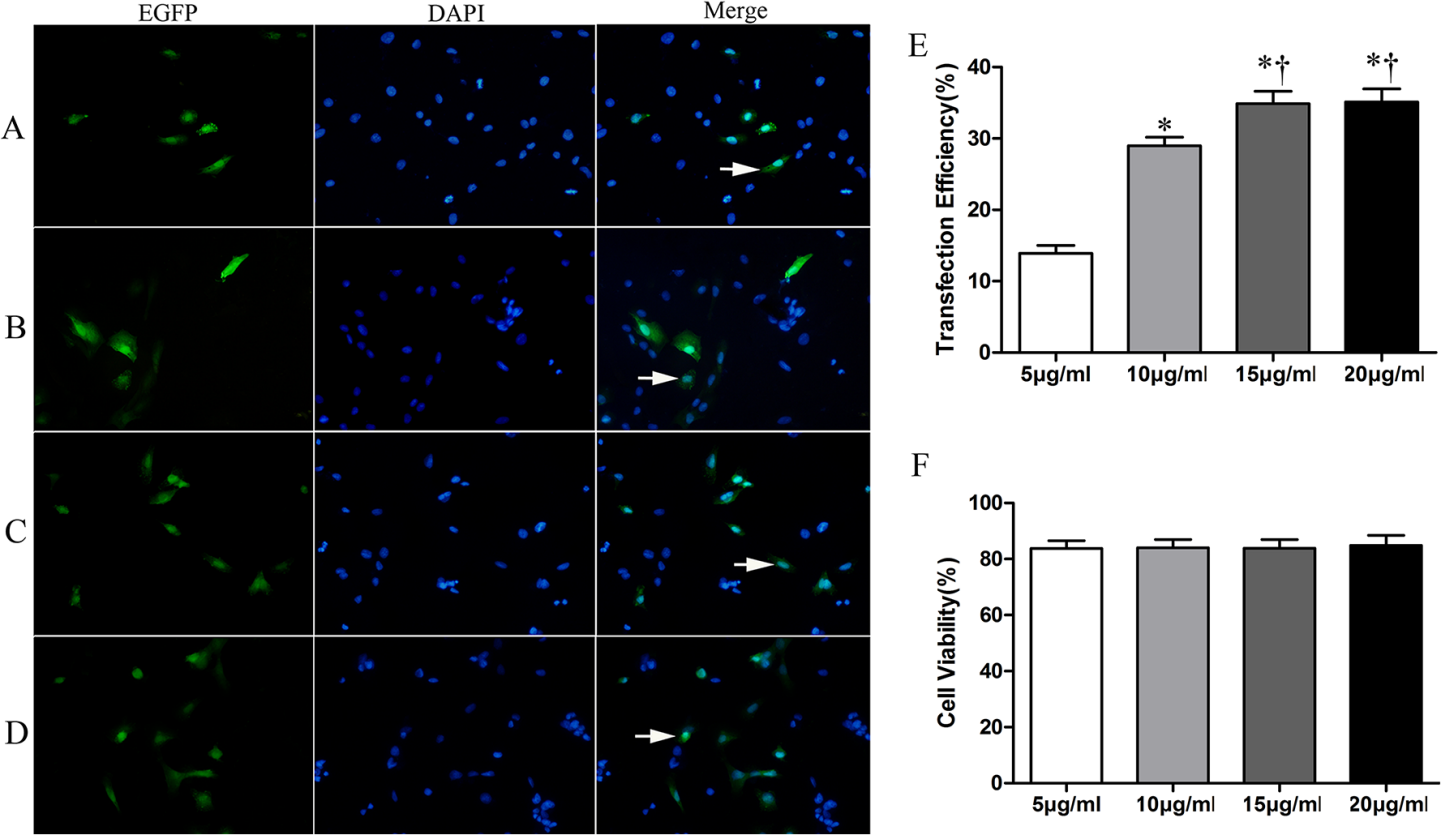
The effect of PC on transfection efficiency and cell viability. A-D showing EGFP expression at PC 5 μg/ml,10 μg/ml,15 μg/ml, 20 μg/ml in sequence. E showing transfection efficiency at different PC with when MC (30%), AI (1.5 W/cm^2^), and ET(30 s) were fixed. F cell viability at different PC with when MC (30%), AI (1.5 W/cm^2^), and ET (30 s) were fixed. Indication: Symbols indicate significant differences (*P*< 0.05) from ET of 5 μg/ml(*) or 10μg/ml (†).

Finally, we fixed MC (300 μl/ml), AI (1.5 W/cm^2^), and ET (30 s), and varied the PC. The cell transfection efficiency was shown in Fig 4A–4D at different PC when MC(300 μl/ml), AI (1.5 W/cm^2^), and ET(30 s) were fixed. EGFP transfection efficiency was significantly increased when the PC was increased up to15 μg/ml (*P*<0.05) (Fig 4E). Increasing PC did not affect the cell viability (Fig 4F).

### The silencing effect of shPHD2 after its transfection mediated by UTMD and PEI

**Fig 5:**
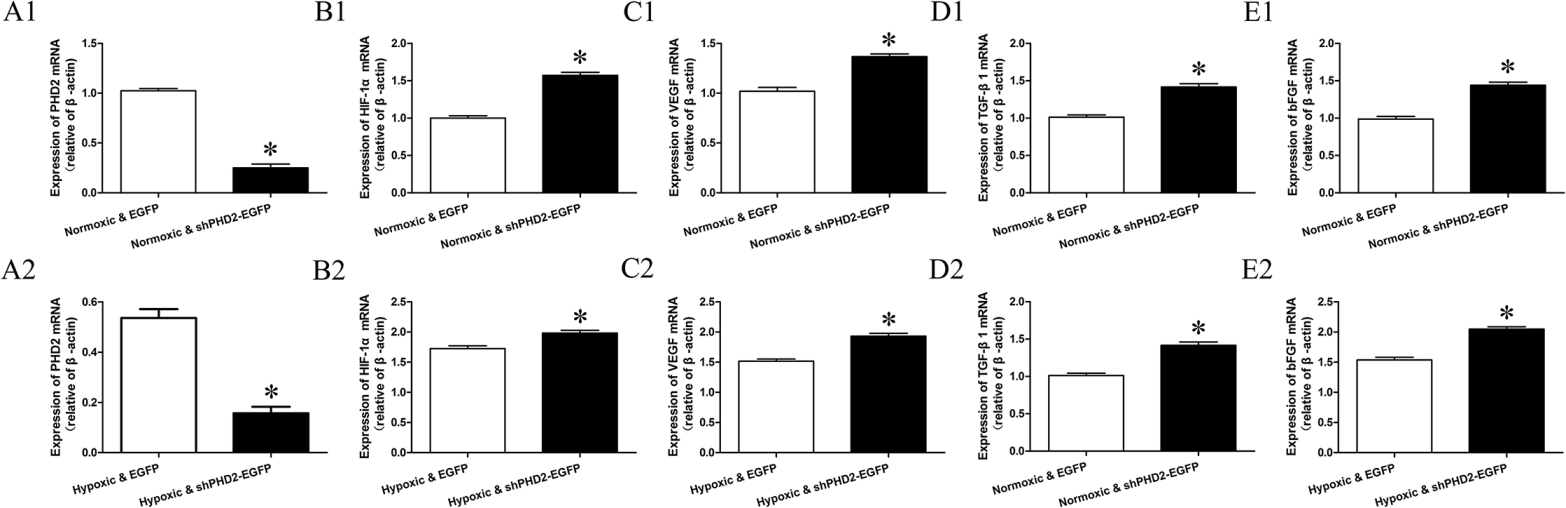
Effects of shPHD2 delivery on PHD2, HIF-1a, VEGF, bFGF and TGF-β1 mRNA and protein expression in H9C2 cells. A-E: Expression of PHD2, HIF-1,VEGF, TGF-β1and bFGF was determined by quantitative polymerase chain reaction(qPCR) in H9C2 cells of Normoxic & EGFP, Normoxic & shPHD2-EGFP, Hypoxic & EGFP and Hypoxic & shPHD2-EGFP group presented in the bar graph (asterisk indicates *P*-value<0.05).

**Fig 6:**
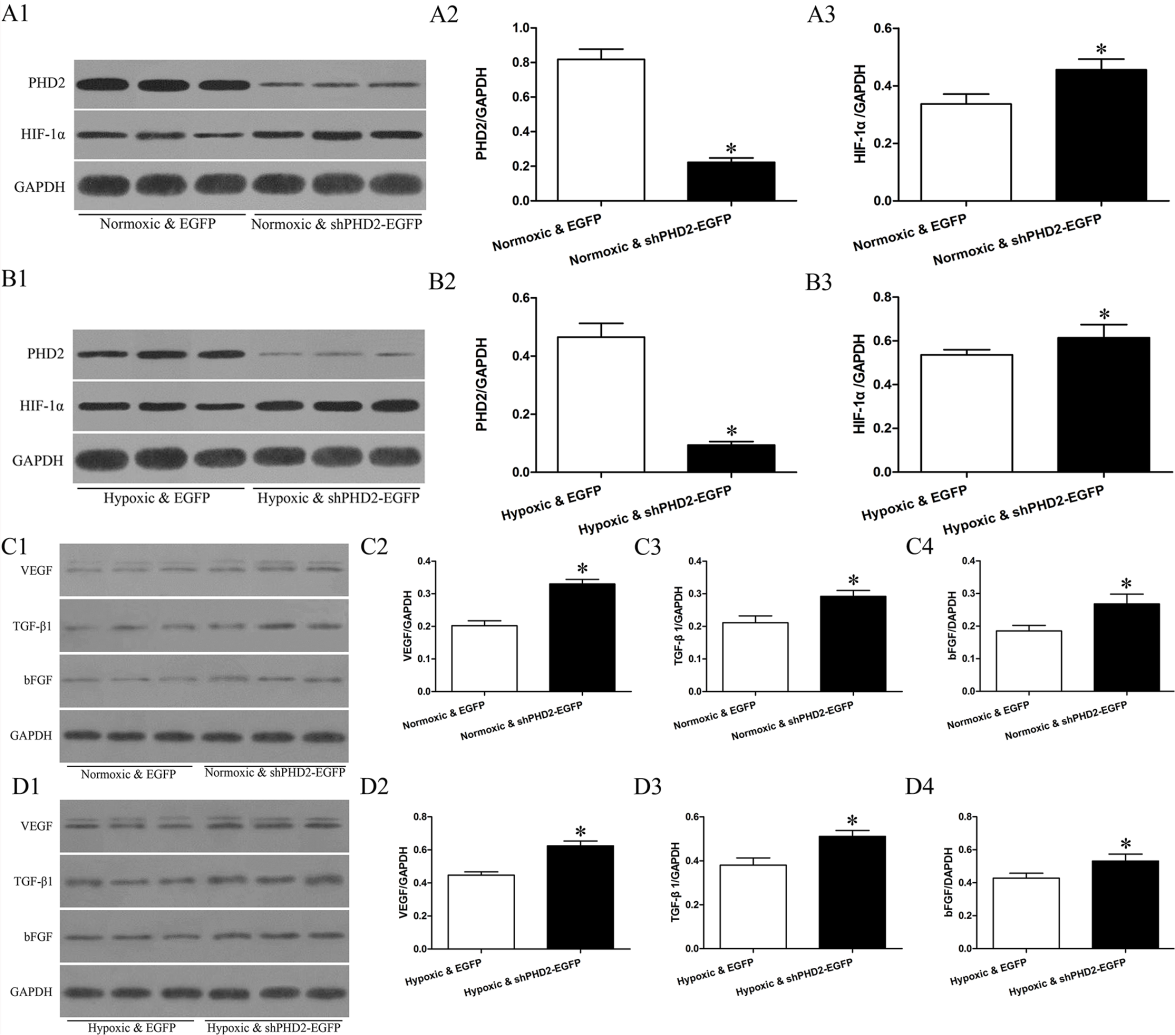
Effects of shPHD2 delivery on PHD2, HIF-1a,VEGF, bFGF and TGF-β1 protein expression in H9C2 cells. A1, B1, C1 and D1: Representative Western-blot, showing bands of PHD2, HIF-1α,VEGF, bFGF and TGF-β1 in myocardial specimens collected from the indicated groups (asterisk indicates *P*—value<0.05). A2-A3,B2-B3,C2-C4 and D2-D4:Densitometric measurements of the PHD2, HIF-1α, VEGF, bFGF and TGF-β1 to GAPDH and presented in the bar graph (asterisk indicates *P*-value<0.05). Indication: Normoxic & EGFP: normoxic H9C2 cells transfected with EGFP plasmid; Normoxic & shPHD2-EGFP: normoxic H9C2 cells transfected with shPHD2-EGFP plasmid; Hypoxic & EGFP: hypoxic H9C2 cells transfected with EGFP plasmid; Hypoxic & shPHD2-EGFP: hypoxic H9C2 cells transfected with shPHD2-EGFP plasmid.

mRNA levels of PHD2, HIF-1α, VEGF, bFGF and TGF-β1 mRNA in Normoxic & EGFP, Normoxic & shPHD2-EGFP, Hypoxic & EGFP and Hypoxic & shPHD2-EGFP group are shown in Fig 5A–5E. Compared with Normoxic & EGFP group, the expression of PHD2 mRNA in Normoxic & shPHD2-EGFP group was reduced. Compared with Hypoxic & EGFP group, the expression of PHD2 mRNA in Hypoxic & shPHD2-EGFP group was significantly reduced. However, the expression levels of HIF-1α, VEGF, bFGF and TGF-β1 mRNA were increased in contrast with the expression of PHD2 mRNA in Normoxic & EGFP, Normoxic & shPHD2-EGFP, Hypoxic & EGFP and Hypoxic & shPHD2-EGFP group. The expression levels of PHD2, HIF-1α, VEGF, bFGF and TGF-β1 protein correlated with corresponding mRNA (Fig 6A–6D).

## Discussion

In recent years, ultrasonic medicine experienced rapid development in the field of diagnosis as well as treatment. Its potential application in gene therapy is attracting attention. Microbubbles and US have been investigated as a method to improve the transfection efficiency of nonviral delivery systems [13–16]. Significant physical effects can be produced during gene/drug delivery into cells in vitro or vivo mediated by US. These consist mainly of thermal effect and cavitation; the latter plays an important role in gene delivery. Sonoporation refers to the formation of small pores in cell membranes, which allow large gene fragment from the surrounding medium to enter into the target cells or tissue, without causing damage [17]. Microbubbles, by acting as cavitation nuclei, applied in combination with US, are thought to potentiate this effect and potentially useful for gene delivery via UTMD [18–20]. However, the transfection efficiency based on UTMD alone was low. PEI, as one of the most effective poly-cationic gene vectors, can condense plasmids DNA into electrostatic complexes, protect the plasmids against nucleases, and enhance the endocytosis and endosomal release of plasmids DNA [21–25]. Studies showed that when UTMD was combined with PEI, the transfection efficiency could be significantly improved, thus providing a new strategy for gene therapy [26–27]. In our experiment, the expressing level of EGFP in E group was higher than other groups, which was consistent with previous findings [26–27].

In theory, ultrasonic irradiation can promote gene delivery into target cells and tissue. However, it has been difficult to determine optimal irradiation parameters because different laboratory conditions. Transfection efficiency is associated with many factors. Different microbubbles demand different ultrasonic parameters during gene transfection. Research has shown that gene could be delivered into mice skeletal muscle by Optison microbubbles without ultrasonic irradiation and by SonoVue only when combined with ultrasonic irradiation [28]. Some literature reported that cells and tissues could be damaged under transient cavitation when gene transfection mediated by microbubbleis combined with high energy US waves [29–30]. Therefore, not only gene, microbubble and cell types are important factors to consider, but also cellular damage. The results of our study demonstrated that the gene transfection efficiency and cell viability were linked with AI, MC, ET and PC.

In our experiment, with the AI and MC increasing, cell transfection efficiency increased during gene transfection when frequency(1MHZ), duty cycle(20%), ET(30 s) and PC(5 μg/ml) were fixed. However, the cell transfection efficiency decreased significantly when AI was over 1.5 Wcm^2^ and MC was over 300μl/ml. US and microbubble not only could prompt gene transfection by increasing membrane permeability, but also cause cell apoptosis because of cavitation effect. In other words, cell transfection efficiency would increase when increasing AI and MC. At same time, it would cause a lot of cells death.

A previous report found that cell viability decreased significantly when the ET changed from 1 s to 60 s. The cell survival rate was 99% and 5% respectively. The transfection efficiency was highest when the ET was 20 s (AI 2.0 W/cm^2^). However, the cell survival rate was only 64%[31]. In our experiment, cell transfection efficiency rose at first and then went down. Meanwhile, cell viability remained relatively unchanged, consistent with another report [32]. Therefore, the optimal ET must be determined so cell transfection efficiency is optimized without excessive cytotoxicity. In our study, the optimal ET was 30 s.

Cell transfection efficiency remained unchanged after initially increasing with an increase in PC. However, cell viability did not change with the increase of PC. In our study, the optimal PC was 15μg/ml, which was in keeping with one report [33]. Therefore, sufficient amount of DNA was necessary for high transfection efficiency.

RNA interference (RNAi) is a process through which a double-stranded RNA (dsRNA) induces the sequence-specific post transcriptional silencing of a gene. It has a high degree of effectiveness and specificity if the delivery problem can be addressed. RNAi has been a useful tool of gene function analysis [10]. At the same time, the effect of short hairpin RNA (shRNA) interference plasmid is potentially superior to synthetic chemical small interfering RNA (siRNA) if delivered into the cellular nucleus. HIF-1α is known to control the expression of over 60 genes that affect cell survival and metabolism in adverse conditions, including vascular endothelial growth factor, fibroblast growth factor, insulin-like growth factor, erythropoietin, and nitric oxide synthase among others [10, 34]. Unfortunately, HIF-1α has a biological half-life of only approximately 5 minutes under normoxic condition [34]. This is because during normoxic condition, HIF-1α is hydroxylated by oxygen-dependent prolyl hydroxylase-2 (PHD2), ubiquitinated, and subsequently degraded. A study showed that the HIF-1α degradation could be inhibited through short hairpin RNA (shRNA) knockdown of PHD2 [10]. In this article, we demonstrated that shPHD2 plasmid can be delivered into H9C2 cells mediated by UTMD and PRI and HIF-1α degradation could be inhibited after shPHD2 transfection. In our experiment, compared with Normoxic & EGFP, the PHD2 mRNA level of Normoxic & shPHD2-EGFP group was decreased. The PHD2 level in Hypoxic & shPHD2-EGFP was lower than Hypoxic & EGFP group. However, the expression of HIF-1α, VEGF, bFGF and TGF-β1 mRNA was increased, in contrast to that of PHD2. The expression levels of PHD2, HIF-1α, VEGF, bFGF and TGF-β1 protein were consistent with corresponding mRNA.

## Conclusions

Gene transfection mediated by UTMD is highly influenced by the parameters, such as AI, MC, ET and PC. Based on our experiments, the AI of 1.5 W/cm^2^, a microbubbles concentration of 300 μl/ml, an ET of 30 s and a PC of 15 μg/ml were optimal transfection parameters. PHD2 could be down-regulated significantly after shPHD2 transfection into H9C2 cells mediated by UTMD and PEI. At the same time, HIF-1α and downstream angiogenesis factors of VEGF, TGF-β and bFGF were up-regulated. These data lays a foundation for future development of an ischemic cardiomyopathy shRNA therapy. Further in vivo studies in a rat model is warranted.
